# Antihyperglycaemic Activity of Standardised Ethanolic Extract of *Swietenia macrophylla* King Seeds on Goto-Kakizaki Type 2 Diabetic Rats

**DOI:** 10.21315/tlsr2025.36.2.5

**Published:** 2025-07-31

**Authors:** Meyyammai Swaminathan, Mariam Ahmad, Khairul Niza Abdul Razak, Nor Adlin Yusoff, Gabriel Akyirem Akowuah, Elaine Hui-Chien Lee, Syed Azhar Syed Sulaiman, Mun Fei Yam, Faradianna E. Lokman, Sue Hay Chan, Bey Hing Goh, Vikneswaran Murugaiyah

**Affiliations:** 1 School of Pharmaceutical Sciences, Universiti Sains Malaysia, 11800 USM Pulau Pinang, Malaysia; 2 Advanced Medical and Dental Institute, Universiti Sains Malaysia, 13200 Kepala Batas, Pulau Pinang, Malaysia; 3 School of Pharmacy, Monash University, 47500 Subang Jaya, Selangor, Malaysia; 4 Department of Diabetes, Cardiovascular, Diabetes and Nutrition Research Centre, Institute for Medical Research, 50588 Kuala Lumpur, Malaysia; 5 Usains Biomics Laboratory, Universiti Sains Malaysia, 11800 USM Pulau Pinang, Malaysia; 6 School of Medical and Life Sciences, Sunway University, Sunway City, 47500 Selangor, Malaysia; 7 College of Pharmaceutical Sciences, Zhejiang University, Hangzhou 310058, China; 8 Centre for Drug Research, Universiti Sains Malaysia, 11800 USM Pulau Pinang, Malaysia

**Keywords:** Antihyperglycaemic, Goto-Kakizaki (GK) rats, Limonoids, *Swietenia macrophylla*, Type 2 Diabetes mellitus (T2DM), Antihiperglisemik, Tikus Goto-Kakizaki (GK), Limonoid, *Swietenia macrophylla*, *Diabetes mellitus* Jenis 2 (T2DM)

## Abstract

*Swietenia macrophylla* (*S. macrophylla*), commonly known as “sky fruit”, belongs to the Meliaceae family and is predominantly distributed in the neotropical areas of Central America, Southern Asia and the Pacific region. The plant has a rich tradition of being utilised for its anti-diabetic properties and other health benefits. This study focused on the *S. macrophylla* seeds ethanolic extract (SMEE) to explore its antihyperglycemic effects in Goto-Kakizaki (GK) Type 2 diabetic rats. Bioactive compounds were extracted using maceration, and a reversed-phase high-performance liquid chromatography (RPHPLC) method was validated to separate two limonoids, swietenine and 3,6-O,O diacetyl swietenolide, from the extracts. The 500 mg/kg SMEE dosage significantly reduced fasting blood glucose levels, making it the selected treatment dose. The SMEE group consistently improved glucose regulation during oral glucose tolerance test (OGTT) on the first (9.88 ± 0.69 mmol/L) and eighth (6.12 ± 0.30 mmol/L) days, showing lower initial fasting blood glucose levels. The RP-HPLC method validation confirmed high linearity, ensuring precise quantification within the 1.56 μg/mL to 200 μg/mL range for swietenine and 3,6-O,O diacetyl swietenolide. The content of these compounds in 1 mg of SMEE was determined as 27.5 μg (2.75%) and 14.53 μg (1.45%), respectively. This study provides robust evidence supporting the antihyperglycaemic properties of *S. macrophylla* seeds. Future studies could evaluate the long-term metabolic effects of *S. macrophylla* extract on glucose metabolism, oxidative stress and liver function.

## Abstrak

*Swietenia macrophylla* (*S. macrophylla*), yang lazimnya dikenali sebagai “buah tunjuk langit”, tergolong dalam keluarga Meliaceae dan banyak ditemui di kawasan neotropika di Amerika Tengah, Asia Selatan dan rantau Pasifik. Tumbuhan ini secara tradisinya digunakan untuk kesan anti-diabetesnya dan faedah kesihatan selainnya. Penyelidikan ini telah memfokuskan terhadap ekstrak etanol biji *S. macrophylla* (SMEE) bagi mengkaji kesan antihiperglisemianya pada tikus diabetes jenis 2 Goto-Kakizaki (GK). Kaedah maserasi digunakan untuk mengekstrak sebatian bioaktif, dan kaedah kromatografi cecair berprestasi tinggi-fasa berbalik (RP-HPLC) dioptimumkan untuk mengasingkan dua limonoid, swietenine dan 3,6-O,O diacetyl swietenolide. Dos SMEE 500 mg/kg telah menurunkan paras glukosa darah puasa dengan ketara, menjadikannya dos rawatan yang dipilih. Kumpulan SMEE secara konsisten menambahbaik pengawalaturan glukosa semasa ujian toleransi glukosa oral (OGTT) pada hari pertama (9.88 ± 0.69 mmol/L) dan hari kelapan, (6.12 ± 0.30 mmol/L) dengan menunjukkan paras glukosa darah berpuasa awal yang lebih rendah. Penentusahan kaedah RP-HPLC mengesahkan kelinearan yang tinggi, memastikan kuantifikasi yang tepat dalam julat 1.56 μg/mL hingga 200 μg/mL untuk swietenine dan 3,6-O,O diacetyl swietenolide. Kandungan sebatian ini dalam 1 mg SMEE ditentukan sebagai 27.5 μg (2.75%) dan 14.53 μg (1.45%), masing-masing. Kajian ini memberikan bukti kukuh yang menyokong sifat antihiperglisemia biji *S. macrophylla*. Kajian masa depan boleh menilai kesan jangka panjang ekstrak *S. macrophylla* terhadap metabolisme glukosa, tekanan oksidatif dan fungsi hati.

**Highlights**

- Limonoids extracted from *Swietenia macrophylla* seeds exhibited antihyperglycaemic effects in diabetic rats like those observed in the positive control group.
- This offers additional confirmation of the antihyperglycaemic properties of the plant extract, especially in a Type 2 diabetic animal model that closely emulates human responses, marking the first instance of such validation.
- The RP-HPLC method is strongly recommended for concurrently identifying and quantifying limonoids in extracts derived from *Swietenia macrophylla*.

## INTRODUCTION

Type 2 diabetes mellitus (T2DM) is a chronic metabolic disorder characterised by disruption of carbohydrate, lipid and protein metabolism, resulting in persistent hyperglycaemia. This condition primarily results from diminishing insulin secretion by pancreatic β cells, impaired insulin action, or a combination of both, known as insulin metabolic syndromes (DeFronzo *et al*. 2015). Diabetic individuals are also at an increased risk of health complications and mortality due to diabetes-related complications (Dahlstrom & Sandholm 2017; Zambrana *et al.* 2018). Generally, the primary goal of all diabetes therapy and management is to maintain blood glucose levels within a healthy range, typically achieved through the use of oral hypoglycaemic agents, insulin and lifestyle modifications. Oral antidiabetic medications can be categorised into four main groups: insulin secretagogues, biguanides, thiazolidinediones and alpha-glucosidase inhibitors, all of which have been extensively utilised (WHO 2016). All of these medication classes operate via distinct mechanisms of action, such as stimulating insulin secretion, reducing hepatic gluconeogenesis, increasing insulin receptor sensitivity and delaying carbohydrate digestion and absorption (Aralelimath & Bhise 2012). However, these treatments often come with various side effects such as hypoglycaemia and weight gain. Risk of severe side effects such as lactic acidosis in patients with renal or hepatic impairment, acute congestive heart failure, sepsis, dehydration and excessive alcohol intake, can compromise glucose control and adherence to treatment, complicating diabetes management (Corathers *et al.* 2013). There is a pressing need for novel therapeutic strategies that minimise adverse effects and enhance patient adherence to manage the progression of this metabolic condition. Ideally, these potential treatments should aim to improve insulin sensitivity, prevent the failure of pancreatic β-cells in T2DM, mitigate or reverse microvascular and macrovascular complications, and have fewer adverse effects (DeFronzo *et al.* 2015; Zambrana *et al.* 2018).

*Swietenia*, a genus of tropical trees classified within the Meliaceae family (Brown *et al.* 2003), is known for its majestic, tall evergreen hardwood trees with winged seeds and anthers located between the teeth of the stamen tube. The name “macrophylla” is derived from the Greek words “makros” (meaning “big”) and “phyllon” (meaning “leaf”), signifying plants with exceptionally large leaves (National Park, 2022). One such species within this genus is *Swietenia macrophylla* King (Family: Meliaceae), commonly referred to as “big leaf mahogany” or “sky fruit.” *S. macrophylla* is native to Central America, South America, and India, but it has been introduced and cultivated in numerous tropical countries such as the Philippines, Malaysia, Singapore, Myanmar and Indonesia (Brown *et al.* 2003; Lin & Mon 2020).

In Malaysia, it is known as “pokok buah tunjuk langit” due to the unique upward growth of its fruit. Traditional knowledge highlights the detoxifying and immune-boosting properties of *S. macrophylla* (Eid *et al.* 2013), and local healers in East Midnapore and West Bengal, India, have a long history of using *S. macrophylla* seeds to address diarrhoea (Maiti *et al.* 2007). Furthermore, this plant has exhibited effectiveness in treating various ailments, including malaria, skin conditions, fever, hypertension and tuberculosis. It serves numerous medicinal purposes as a tonic, astringent, purgative and depurative (Nilugal *et al.* 2017). *Swietenia macrophylla* is also known to be traditionally consumed as anti-diabetic medicine by the olden folklore. Several studies have reported the utilisation of *S. macrophylla*’s leaf extract, seed oil (Eid *et al*. 2013; Nilugal *et al.* 2017) and seeds (Goh & Kadir 2011; Hashim *et al.* 2013) for their anti-diabetic properties.

Several tetranotriterpenoids or limonoids, notably swietenine and 3,6-O,O-diacetyl swietenolide, have been successfully isolated and identified from *S. macrophylla* seeds (Falah *et al.* 2008). Swietenine has been regarded as the primary phytochemical responsible for its anti-diabetic properties, as it promotes glucose utilisation, activates PPARγ, inhibits superoxide generation, and hinders nitric oxide production (Dewanjee *et al.* 2020). Furthermore, both swietenine and 3,6-O,O-diacetyl swietenolide facilitate the increased translocation of GLUT4 to the plasma membrane, thereby enhancing glucose absorption by muscle cells. These limonoids have demonstrated significant anti-diabetic potential with minimal side effects related to weight gain (Lau *et al.* 2015).

Our research seeks to determine the presence of swietenine and 3,6-O,Odiacetyl swietenolide in *S. macrophylla* seed ethanolic extract (SMEE). To accomplish this, we have developed and validated a simple, accurate and rapid reversed-phase HPLC-UV vis method for the SMEE standardisation, utilising swietenine and 3,6-O,O-diacetyl swietenolide as the marker compounds. Building upon previous findings regarding *S. macrophylla* anti-diabetic attributes, we aim to investigate the antihyperglycaemic activity of standardised ethanol extract derived from *S. macrophylla* seed in Goto-Kakizaki (GK) Type 2 diabetic rats. This study aimed to shed light on the extract’s impact on a genuine T2DM disease model. Goto-Kakizaki rats are an excellent representation of non-obese Type 2 diabetes animal models, as they possess distinctive characteristics and functional aspects closely resembling those of human T2DM patients. The outcomes of this investigation will provide valuable evidence further corroborating the antidiabetic properties of *S. macrophylla* seed extract.

## MATERIALS AND METHODS

### Chemicals

Glibenclamide, Tween 80 [C_6_H_124_O_26_] and Phosphate Buffered Saline (PBS) tablets were purchased from Sigma-Aldrich (St. Louis, MO, USA); D(+)-Glucose Anhydrous [C_5_H_12_O_5_] was purchased from Bendosen (Kuala Lumpur, Malaysia); ethanol 95 % was purchased from Fine Chemicals (London, UK); Enzyme-Linked Immunosorbent Assay (ELISA) Kit was purchased from Cloud-Clone Corp (Katy, TX, USA). All reagents were of analytical grade.

#### Preparation of extract

The seeds of *S. macrophylla* were gathered in Kepala Batas (5.3359° N, 100.2837° E), Penang, Malaysia and their identification was conducted by Associate Professor Dr. Rahmad Zakaria from the Herbarium Unit, School of Biological Sciences, Universiti Sains Malaysia (USM). A voucher specimen (11707) has been securely deposited at the Herbarium of the School of Biological Sciences, USM.

A total of 50 g of dried *S. macrophylla* seed powder was immersed in 2 L of 95% ethanol. The extraction process was carried out through maceration and lasted for 48 h, with the temperature maintained at 45°C in a water bath. At two-hour intervals, the mixture was thoroughly agitated to ensure the effective permeation of the solvent through the seed material. Subsequently, the extract was filtered using Whatman’s filter paper and then concentrated using a vacuum rotary evaporator (Buchi Labortechnik, Flawil, Switzerland). The concentrated extract was further subjected to lyophilisation, which was accomplished using a freeze dryer (Labconco Corporation, Kansas City, Missouri, USA), for 5 days. The resultant lyophilised extract was stored in a glass sample container and kept refrigerated at temperatures ranging from 0°C to −4°C for later use in the designated experiments.

### Plant Extract Standardisation

#### Standards, samples and chemicals

Standards, swietenine and 3,6-O,O-diacetyl swietenolide were solubilised in pure methanol to attain individual stock solutions with a concentration of 1 mg/mL each. Subsequently, a combined standard stock solution was created by mixing these individual standard stocks, resulting in a concentration of 0.4 mg/mL. The *S. macrophylla* extract was also dissolved in pure methanol to reach a final concentration of 1 mg/mL. Before proceeding with the analysis, the solution underwent filtration using 0.20 μm nylon filters.

HPLC-grade methanol and acetonitrile were acquired from Merck Life Sciences Private Limited (Vikhroli, Mumbai). For HPLC purposes, deionised water was meticulously prepared utilising the Ultra-pure Water Scholar UV, Model: NEX UP 1000, by Human Corp. in Seoul, Korea.

#### HPLC instrumentation and chromatographic conditions

For the quantification of two limonoids (swietenine and 3,6-O,O-diacetyl swietenolide) in *S. macrophylla* seed extracts, a high-performance liquid chromatography (HPLC) system from Shimadzu, Japan, was employed. This HPLC system was equipped with a quaternary pump, an auto-sampler, a vacuum degasser, an automatic thermostatic column compartment, a UV detector and data processing software known as Lab Solutions. The chromatography separation was carried out on Agilent TC-C18 column (250 mm × 4.6 mm, 5 μm) [Agilent Technologies, Santa Clara, USA] at a column temperature of 30°C, low-pressure gradient and flow rate of 1.0 mL/min using water (A), transitioning from 70% to 10%, and acetonitrile (B), transitioning from 30% to 90% for 30 min, as mobile phase. Column regeneration was continued thereafter for 5 min, totalling the complete cycle to 35 min (Schefer *et al.* 2006). The injection volume was 20 μL and the detection wavelength was set at 220 nm.

#### Limit of detection (LOD), limit of quantification (LOQ) and linearity

The Limit of Detection (LOD) represents the minimum quantity of an analyte within a sample that can be identified, although it may not be precisely quantified under the given experimental parameters. Typically, the detection limit is stated in terms of the analyte’s concentration in the sample. The LOD can be determined through various methods, such as visual assessment, the signal-to-noise ratio, or calculations involving the standard deviation of the response (s) and the slope (S).

The Limit of Quantification (LOQ) signifies the minimum analyte quantity in a sample that can be reliably and accurately measured under the specified experimental conditions. It is typically expressed as the analyte’s concentration within the sample. In this study, both LOD and LOQ are determined by visually evaluating the peaks in the data.

To assess linearity, five replicates of various known concentrations from a mixed standard solution were examined. To establish linearity, a serial dilution was carried out, commencing at 400 μg/mL from the mixed standard stock solution and employing methanol to attain concentrations ranging from 1.56 μg/mL to 200 μg/mL. A calibration curve was generated by plotting the peak area against the concentration, and linear regression equations were calculated. Additionally, the correlation coefficient (R2) was determined.

### Validation of the Method

Validation of an analytical method is crucial to establish its accuracy, precision and robustness. The method validation was conducted following the guidelines set forth by the International Conference of Harmonisation (ICH). Specifically, the method was validated for specificity, recovery, and precision.

Stock solutions were prepared as 1.02 mg of swietenine in 1.02 mL methanol and 1.09 mg of 3,6-O,O-diacetyl swietenolide in 1.09 mL methanol, correspondingly. The concentrations of each stock solution were 1 mg/mL each. Subsequently, 0.4 mL of swietenine (1 mg/mL) and 0.4 mL of 3,6-O,O diacetyl swietenolide (1 mg/mL) were combined with 0.2 mL of methanol to form a mixed standards stock solution with a concentration of 0.4 mg/mL. From this mixed standard stock solution, a range of working standard solutions (at concentrations of 50 mg/mL, 12.5 mg/mL and 1.56 mg/mL) were prepared by dilution with methanol.

These working standard solutions were utilised to assess the method’s recovery, within-day and between-day accuracy, and precision. Separate standard curves were generated for each day of analysis. Within-day accuracy and precision were determined for each compound at four different concentrations, with each concentration being replicated five times within a single day. Between-day values were assessed over six consecutive days. To determine the extraction recovery, the pulverised plant matrix was spiked with the working standard solutions of each compound. The spiked samples were then subjected to extraction as detailed earlier. The extraction recovery for each compound was calculated as a percentage, representing the concentration of the spiked compound obtained after extraction relative to the equivalent amount without extraction.

#### Specificity

The specificity of the method was determined by subjecting standard substances against potential interferences. This evaluation involved injecting six replicates of the 100% test (sample) solution.

#### Precision

Precision was assessed by the examination of repeatability (intraday) and intermediate (interday) precision. The precision of an analytical method is usually expressed as the percentage relative standard deviation (%RSD)/coefficient of variation (CV) derived from a series of measurements (Naveen *et al.* 2018). In this study, precision was determined by calculating the %RSD at three distinct concentrations: 1.56 μg/mL, 12.5 μg/mL and 50 μg/mL (representing low, medium and high concentrations) of mixed standard solutions. Repeatability was tested five times within the same day, while intermediate precision was assessed over a period of 5 days.

#### Accuracy

To assess the method’s accuracy, recovery studies were conducted by adding three distinct concentrations (1.56 μg/mL, 12.5 μg/mL and 50 μg/mL, representing low, medium and high concentrations, respectively) of mixed standard stock solutions into separate samples of SMEE (1 mg/mL). These spiked samples underwent triplicate injections for examination.

The Percentage of Recovery was calculated using the following formula:


% recovery=(b-a)/c×100

where a = the amount of drug found in the sample before the addition of the standard drug, b = the amount of drug found after the addition of the standard drug and c = is the amount of standard drug added.

#### Antihyperglycaemic activity

The antihyperglycaemic effects of SMEE were evaluated in GK Type 2 diabetic rats, with normal Sprague-Dawley (SD) rats serving as the control group.

### Experimental Animals

In this study, healthy adult male GK Type 2 diabetic rats were obtained from CLEA Japan Inc. (Tokyo, Japan), while adult male SD rats were sourced from the Animal Research and Service Centre (ARASC) of Universiti Sains Malaysia. The rats used in the experiment weighed between 160 g to 250 g and were 12 weeks to 16 weeks old. All the animals were housed in plastic cages and provided with commercial feed and water *ad libitum*. They were kept in a standard, well-ventilated environment with conditions maintained at 24 ± 1°C and a light/dark cycle of 12 h each throughout the study. The study protocol received approval from the Institutional Animal Care and Use Committee (IACUC) at Universiti Sains Malaysia under the approval number USM/IACUC/2018/(112)(923).

#### Experimental groups

In this study, a total of 24 adult male GK rats and 6 SD rats were included. The rats were chosen at random and sorted into groups (NC, DC, GB, SMEE 250 and SMEE 500) as follows: Group NC, which comprised 6 normal SD rats served as the negative control. They received a vehicle solution (4% Tween 80; 10 mL/kg). Group DC, consisting of 6 GK Type 2 diabetic rats served as the negative control for the disease. These rats were diabetic and received the same vehicle solution (4% Tween 80; 10 mL/kg). Group GB, comprises 6 GK diabetic rats and served as the positive control group. These rats received Glibenclamide (10 mg/kg b.w.). Group SMEE 250, consisting of 6 GK diabetic rats, served as a treatment group. These rats received SMEE at a dose of 250 mg/kg b.w. Group SMEE 500, which also included 6 GK diabetic rats served as a treatment group. These rats received SMEE at a dose of 500 mg/kg b.w. All treatments were administered twice daily via oral gavage, and the chosen doses were based on findings from similar studies (Hashim *et al.* 2013; Kalaivanan & Pugalendi 2011b).

#### Antihyperglycaemic effect of SMEE in GK Type 2 diabetic rats

Before the collection of fasting blood samples, all animals in groups NC, DC, GB 10, SMEE 250 and SMEE 500 underwent a 10 h fasting period. Blood samples were obtained at time points 0 (before treatment), 1 h, 2 h, 3 h, 5 h and 7 h after treatment initiation using the tail prick method. The respective treatments, as previously detailed in Section 2.4.2, were freshly prepared and administered to the rats via oral gavage. Fasting blood glucose levels (FBGL) were assessed using the Accu-Chek Performa Clinical Glucose meter (Roche, USA). The same treatment regimen was maintained over 8 days, with treatments administered twice daily and a 12 h interval between doses (at 8:00 a.m. and 8:00 p.m.). Blood glucose levels were determined on both days 1 and 8 to evaluate the antihyperglycemic effects of SMEE at doses of 250 mg/kg and 500 mg/kg on the diabetic rats.

#### Antihyperglycaemic evaluation of sub-chronic (14 days) treatment of SMEE in GK Type 2 diabetic rats

In the sub-chronic study, the identical groups of diabetic animals mentioned in Section 2.4.3 (groups NC, DC, GB and SMEE 500) maintained their twice-daily oral treatments. The rats’ body weight was monitored daily. Fasting blood glucose levels were assessed in rats that had fasted for 10 h, both before the commencement of treatment on day 8 and upon concluding the 14-day treatment period.

#### Oral Glucose Tolerance Test (OGTT)

Before the commencement of the experiment, all rats from groups NC, DC, GB and SMEE 500 underwent a 10 h fasting period. The weight and FBGL of the rats were measured. Additionally, blood samples were drawn from the rats’ tail veins for a plasma insulin study. Glucose (2 g/kg) was administered to the rats via an intragastric method using oral gavage. Fasting blood glucose levels were monitored at intervals of 30 min, 60 min, 90 min and 120 min following glucose ingestion using the Accu-Chek Performa Clinical Glucose meter (Roche, USA) as previously described. Blood samples were collected for the plasma insulin study at time points 0 min, 30 min and 120 min after glucose ingestion. Insulin levels were determined using a commercially available ELISA Kit (Cloud-Clone Corp. Katy, TX, USA). Following the completion of blood sample collection, the rats were granted unrestricted access to commercial feed and water.

### Statistical Analysis

All outcomes are presented as the mean ± SEM. The antihyperglycaemic FBGL, blood glucose levels, and plasma insulin levels for OGTT investigations, as well as the body weight of rats before and after treatments, were computed using GraphPad Prism version 9.5.1, GraphPad Prism software. The statistical significance of the results was evaluated using one-way or two-way analysis of variance (ANOVA) (*p* < 0.05), followed by the Bonferroni posttest.

## RESULTS

### Preparation of *S. macrophylla* Seed Ethanolic Extract

The achieved yield was 42.27% w/w, and the extract was stored in a glass sample container at temperatures ranging from 0°C to −4°C for future utilisation in the designated experiments.

### Plant Extract Standardisation

#### HPLC method validation and quantification of bioactive compounds

To isolate the two compounds from the solutions (mix compound and SMEE solutions), a combination of water and acetonitrile was employed as mobile phases. Multiple mobile phase compositions were tested, and a satisfactory separation was achieved by implementing a low-pressure gradient elution with a water-to-acetonitrile ratio of 70:30 v/v (A:B) at a flow rate of 1 mL/min. The entire run duration was set at 35 min. The gradient elution involved a change in the acetonitrile concentration [B: 0 to 30 min: 30% to 90%, 30 to 35 min: 90% to 30%] and was employed for quantifying the two compounds.

#### Limit of detection (LOD), limit of quantification (LOQ) and linearity

The calibration curve was generated by plotting the peak area against concentration, covering a range from 1.56 μg/mL to 200 μg/mL. The linearity of these curves was evaluated using linear regression analysis, resulting in the linear regression equations: y = 32937x + 67200 (for swietenine) and y = 19001x + 47777 (for 3,6-O,O diacetyl swietenolide) (Stefanini-Oresic 2022). The correlation coefficients (R^2^) achieved were 0.99 for both compounds respectively. These values indicate a strong fit of the curves, demonstrating excellent linearity for both standards, consistent with the acceptance criteria (R^2^ not less than 0.99) (Stefanini-Oresic 2022).

### Validation of Method

Table 1 depicts precision data for intraday and interday. The calibration curve equations used to calculate concentrations for swietenine are expressed as y = 24539x + 3510.2, while for 3,6-O,O diacetyl swietenolide, the equation is y = 17667x + 2917.4.

In the interday precision analysis (as indicated in Table 1), low relative standard deviation percentages (%RSD) were observed, ranging from 0.75% to 2.99% for swietenine (concentration: 12.50 μg/mL to 200.00 μg/mL), and 0.82% to 2.88% for 3,6-O,O diacetyl swietenolide (concentration: 12.50 μg/mL to 200.00 μg/mL). Slightly elevated %RSD values were noted at the lowest concentration of 1.56 μg/mL for both compounds [swietenine: %RSD = 22.84 and %RSD = 20.74] (International Council for Harmonisation 2019).

In terms of intraday precision analysis (Table 1), the relative standard deviation percentages (%RSD) for peak areas of swietenine standard concentrations (1.56 μg/mL, 12.50 μg/mL, 50 μg/mL and 200 μg/mL) were determined to be 0.62, 0.12, 0.38 and 0.27, respectively. Similarly, for the peak areas of 3,6-O,O diacetyl swietenolide standards within the same concentration range, the %RSD values were 0.48, 0.23, 0.29, and 0.22. In summary, the method established in this study exhibits acceptable intraday precision with %RSD values below 1.0%, and interday precision with %RSD values below 10%. This demonstrates the method’s precision and reproducibility.

The accuracy of an analytical procedure is determined by its ability to closely align test results with the actual values. To determine accuracy, the percentage of the standard (calculated spike concentration) added to the pre-analysed sample (unspiked concentration) was calculated to derive the expected concentration, denoted as C. The concentration of the analyte per millilitre recovered by the assay is labelled as spiked concentration, represented as D. Subsequently, the percentage recovery, which signifies the correspondence between the spiked and predicted analyte concentrations, was calculated and presented in Table 2. The recovery values for the concentrations under examination ranged from 89.56% to 101.41% for swietenine and from 92.44% to 102.67% for 3,6-O,O diacetyl swietenolide. The accuracy values fell within the range of 88.0% to 101.0% for swietenine and 92.00% to 103.00% for 3,6-O,O diacetyl swietenolide.

These findings indicate that the HPLC method employed for the separation and quantification of swietenine and 3,6-O,O diacetyl swietenolide is reliable, repeatable and accurate.

**Figure 1:**
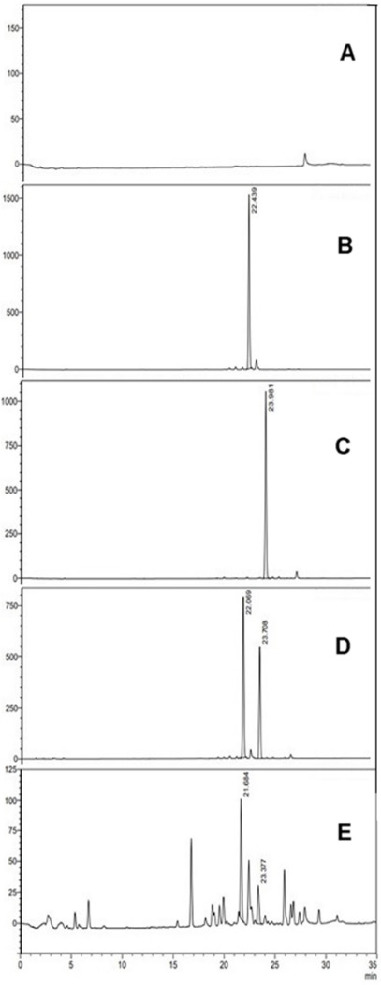
Optimised HPLC chromatograms of blank, (A) methanol, (B) standard compound swietenine and (C) 3,6-O,O diacetyl swietenolide. Peaks observed at retention time t_R_ = 22.44 min and 23.98 min, respectively, for (B) and (C). (D) Mix standard (compound swietenine and 3,6-O,O diacetyl swietenolide, 0.5 mg/mL each). Peaks were observed at 22.07 min and 23.71 min, respectively. (E) SMEE (1 mg/mL) where compounds swietenine and 3,6-O,O diacetyl swietenolide were identified at peaks 21.68 min and 23.38 min, respectively.

Fig. 1 (A–D) illustrates the chromatograms of various solutions, including blank (methanol), swietenine (1 mg/mL), 3,6-O,O diacetyl swietenolide (1 mg/mL), and a mixture of standard compounds swietenine and 3,6-O,O diacetyl swietenolide (0.5 mg/mL each). Fig. 1(E) displays the chromatogram of SMEE (1 mg/mL). Further exploration of the chemical composition of 1 mg/mL of *S. macrophylla* ethanolic extract led to the identification and quantification of swietenine (27.54 μg/mg SMEE) and 3,6-O,O diacetyl swietenolide (14.531 μg/mg SMEE) with retention times of 21.68 min and 23.38 min, respectively. These measured values fall within the range of standard solutions, confirming the method’s specificity (Naveen *et al.* 2018).

#### In-vivo study

The study encompassed optimising treatment dosages, conducting oral glucose tolerance tests (OGTT), monitoring body weight changes and evaluating antihyperglycaemic activity in GK Type 2 diabetic rats.

#### Effects of 250 mg/kg and 500 mg/kg SMEE on fasting blood glucose in GK Type 2 diabetic rats

**Figure 2:**
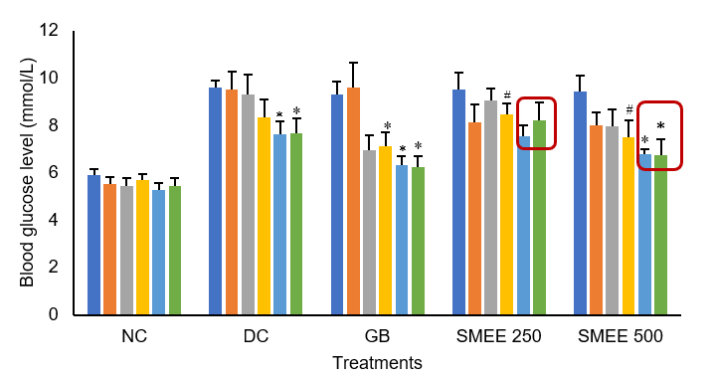
Effects of SMEE 250 mg/kg and 500 mg/kg on fasting blood glucose levels of Type 2 diabetic GK rats. Rats from each group received their respective treatments. Group (NC): SD rats + 4% Tween 80, (DC): GK rats + 4% Tween 80, (GB): GK rats + Glibenclamide (10 mg/kg), (SMEE 250): GK rats + SMEE 250 mg/kg and (SMEE 500): GK + SMEE 500 mg/kg. Symbol (#) represents a significant difference (*p* < 0.05) and (*) represents a significant difference (*p* < 0.001) compared to 0 h of each group.

Fig. 2 depicts the impact of 250 mg/kg and 500 mg/kg SMEE on FBGL of Type 2 diabetic GK rats. Initially, all Type 2 diabetic GK rats in groups DC, GB, SMEE 250 and SMEE 500 exhibited elevated blood glucose levels when compared to the negative control normal rats in group NC at the 0 hour mark. Throughout the study duration, there were no indications of toxicity or recorded fatalities in the rats treated with 250 mg/kg and 500 mg/kg SMEE.

The Type 2 diabetic GK rats in group DC displayed a reduction in blood glucose levels from 0 hours to the 7th hours. This can be attributed to their impaired ability to regulate glucose during fasting. However, it’s important to note that even though there was a downward trend in blood glucose levels, the final FBGL value at the 7th hour remained elevated at 7.67 mmol/L, indicating a sustained hyperglycaemic condition in the rats after 17 h of fasting.

In contrast, group SMEE 250 exhibited inconsistent blood glucose readings at the 5th hour (7.54 mmol/L) and 7th hour (8.22 mmol/L), while group SMEE 500 displayed a continuous decrease in FBGL with sustained lower readings during the same time points (5th hour = 6.780 mmol/L and 7th hour = 6.74 mmol/L). Positive control rats in group GB achieved a significant (*p* < 0.001) reduction in FBGL at the 5th (6.31 mmol/L) and 7th hour (6.27 mmol/L) compared to the initial reading at the start of the experiment. Group SMEE 500 demonstrated similar improvements in FBGL as the positive control, whereas this was not observed in group SMEE 250. Consequently, the 500 mg/kg dose of SMEE was selected for subsequent tests.

#### Sub-chronic (14 days) antihyperglycaemic test in GK Type 2 diabetic rats

**Figure 3:**
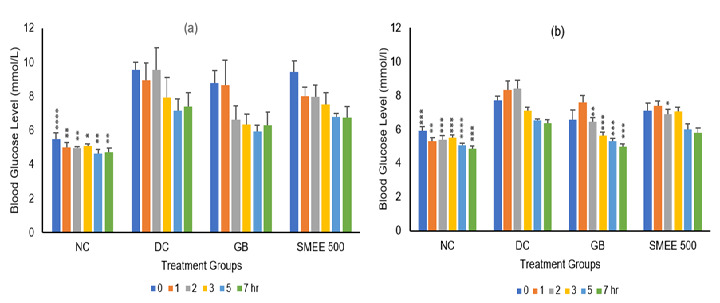
Antihyperglycaemic study on day 1 and 8 (groups NC, DC, GB and SMEE 500) of 14 days of treatment. Group (NC): 4% Tween 80, (DC): 4% Tween 80, (GB): Glibenclamide (10 mg/kg) and (SMEE 500): SMEE 500 mg/kg. Each value represents the mean ± SEM of six rats (*n* = 6). Symbol (*) represents a significant difference (*p* < 0.05), (**) represents a significant difference (*p* < 0.01), (***) represents a significant difference (*p* < 0.001) and (****) represents a significant difference (*p* < 0.0001) when compared with the disease control group (DC) at the same time. Group NC was compared with DC and obtained the mentioned significant glucose reading when compared within the same hour on the same day.

Fig. 3 illustrates the antihyperglycaemic activity of four rat groups (NC, DC, GB and SMEE 500) on days 1 and 8 of the 14-day treatment study. Statistical analysis reveals that GK Type 2 diabetic rats in Group DC exhibit significantly higher FBGL in comparison to the normal rats in Group NC.

In the context of the treatment effects, the positive control (GB – day 8) and the treated group (SMEE 500 – day 8) were compared to the negative control group (DC – day 8) based on the timing of FBGL measurements. Rats in group SMEE 500 – day 8 exhibited significantly lower FBGL at the 2nd hour. Conversely, rats in group GB – day 8 demonstrated significantly lower FBGL levels at the 2nd, 3rd, 5th and 7th hours following their respective treatments.

### Body weight of rats on Days 1 and 15

Table 3 displays the body weights of the rats before the treatment (day 1) and after 14 days of treatment (day 15). Group C rats demonstrated a 9.44% increase in body weight (*p* < 0.05) after the 14-day treatment with 500 mg/kg SMEE.

### Oral Glucose Tolerance Test

Initial blood glucose levels (Table 4) and plasma insulin levels (Table 6) were measured on day 1 of the OGTT before the administration of the control drug or SMEE. Rats in the GB group had significantly lower plasma insulin levels in comparison to rats in the DC group (*p* < 0.001) and SMEE 500 group (*p* < 0.01). Following 14 days of treatment with glibenclamide (10 mg/kg), the positive control rats in the GB group exhibited a significant increase in plasma insulin levels of 0.18 μg/mL, 0.15 μg/mL and 0.08 μg/mL at 0 min, 30 min and 120 min, respectively, after the glucose challenge during the final OGTT on day 15. Higher plasma insulin levels were observed at 0 min, gradually decreasing at 30 min, and dropping even further at 120 min, indicating the positive effect of glibenclamide in enhancing glucose utilisation over a 2-h period (Table 6).

During the OGTT on day 15, plasma insulin levels in rats treated with SMEE (group SMEE 500) were elevated at 0 min, experienced a reduction at 30 min after the glucose challenge, and remained stable up to 120 min (Table 6). This indicates that diabetic rats treated with the positive control (glibenclamide) and SMEE, administered 12 h before the glucose challenge, effectively managed to regulate a gradual decrease in blood glucose levels over a 2-h period after ingestion. The plasma insulin levels in SMEE-treated rats (group SMEE 500) fell within a similar range as those in the GB group and were lower than those in the DC group on day 15 (Table 6).

The rats in the negative group DC showed higher insulin levels at 120 min compared to 30 min, as diabetic rats require more time to metabolise the ingested glucose. These observations indicate that the presence of glibenclamide (10 mg/mL) and SMEE (500 mg/kg) in their respective groups of rats for a period of 12 h played a role in improving the utilisation of glucose (Table 5).

As shown in Table 6, the plasma insulin levels did not differ significantly after repeated oral treatment with glibenclamide 10 mg/kg (GB) or SMEE 500 mg/kg (SMEE 500) in Type 2 diabetic rats. No significant changes in the plasma insulin levels were found between the disease control group (DC) and treated-diabetic rats (SMEE 500), either before or after treatment. On the other note, rats from the GB group displayed increased starting plasma insulin levels of 0.37 ± 0.07 on day 1 to 0.55 ± 0.02 on day 15. Whereas SMEE 500 rats have shown a decrease in starting insulin levels (0.52 ± 0.05 to 0.47 ± 0.05) within the same comparison period. After 14 days of treatment with glibenclamide, it appears that there may be a prolonged biological effect of this hypoglycemic sulfonylurea in regulating plasma insulin level in the bloodstream (Laghmich *et al.* 1999). This suggests that glibenclamide may offer lingering benefits in blood sugar regulation. In contrast, SMEE at a dosage of 500 mg/kg demonstrates decreased plasma insulin level which potentially highlights increased insulin sensitivity, leading to overall better glucose metabolism in the organism, thus less insulin was needed to break down the same amount of glucose ingested. Identification of swietenine and 3,6-O,O diacetyl swietenolide presence in SMEE explains insulin mimicking activity and improved glucose utilisation might have contributed to the reduction in insulin released at 0 h of day 15. To observe significant reactions, it is recommended to do glucose challenge after 1 h to 2 h of treatment ingestion.

## DISCUSSION

Antidiabetic research has traditionally relied on experiments involving streptozotocin (STZ)-induced diabetic rats. A single high dose of STZ injection can lead to the development of Type 1 diabetes by damaging the β-cells in the pancreas (Palmer *et al.* 1998; Wu & Yan 2015). On the other hand, a milder dose of STZ can result in Type 2 diabetes in adult rats (Maiti *et al*. 2005). More recent studies have employed a combination of STZ and nicotinamide administration to create a Type 2 diabetic adult animal model (Pellegrino *et al.* 1998; Yan 2022). To confirm the antihyperglycaemic effects of *S. macrophylla*, it is preferable to assess its impact using the in-vivo method on GK Type 2 diabetic rats.

Goto-Kakizaki rats are widely acknowledged as one of the most suitable non-obese and non-hypertensive animal models for Type 2 diabetes. These rats possess key characteristics that closely resemble those found in human diabetic patients, making them a valuable tool for research (Badole & Jangam 2015). They exhibit consistent glucose intolerance levels and a compromised insulin response to glucose, (Östenson & Efendic 2007), reflecting the inability of beta cells to cope with increased insulin demands. When compared to other animal models, GK rats offer a closer representation of human Type 2 diabetes, especially in terms of polygenic inheritance (Östenson & Efendic 2007), making them an ideal choice for investigating various pathological mechanisms related to Type 2 diabetes.

In this study, we employed glibenclamide, a long-acting sulfonylurea, as a positive control. Glibenclamide operates by stimulating insulin production from the pancreatic beta cells, thereby reducing blood glucose levels in hyperglycaemic conditions (Rajasekaran *et al*. 2005). Furthermore, glibenclamide has been shown to have additional effects, such as a significant increase in Hb, a reduction in HbA1c, and the restoration of liver glycogen levels to normal (Kalpana *et al.* 2011). Our investigation in this study involved comparing the antidiabetic properties of *S. macrophylla’s* seeds to those of glibenclamide in GK rats.

Maiti and colleagues previously reported the hypoglycaemic and hypolipidemic effects of *S. macrophylla* on Type 2 diabetic rats induced by streptozotocin and nicotinamide (Maiti *et al*. 2008). Our research, as documented in our prior work (Hashim *et al*. 2013), has also provided further evidence of the antidiabetic potential of this traditional medicinal plant. Subsequently, we conducted additional studies using *S. macrophylla* to reinforce the claims regarding its anti-diabetic properties. The seeds of *S. macrophylla* were subjected to extraction with 95 % ethanol using the maceration method, resulting in a yield of 42.27% w/w *S. macrophylla* seeds ethanolic extract (SMEE).

*S. macrophylla* seeds have exhibited noteworthy hypoglycaemic potential (Maiti, Dewanjee, Kundu, *et al.* 2007), likely attributed to their insulin-mimicking properties. The seeds of *S. macrophylla* have been shown to enhance peripheral glucose utilisation, and the active hypoglycaemic compound was identified as swietenine through physicochemical and spectrometric analysis (Kadota *et al.* 1990). These seeds have consistently maintained blood glucose levels within the normal range, even in Streptozotocin-induced Type 2 diabetic rats throughout the study period (Hashim *et al.* 2013). Furthermore, the compound 3,6-O,O diacetyl swietenolide facilitated the translocation of GLUT4 to the plasma membrane, thereby increasing glucose uptake by muscle cells. Both of these limonoids have demonstrated significant antidiabetic activity with minimal side effects such as weight gain (Lau *et al.* 2015). Consequently, we have investigated the antidiabetic effects of a standardised ethanolic extract of *S. macrophylla* seeds on GK rats.

Swietenine and 3,6-O,O diacetyl swietenolide, the two limonoids, were detected and measured in the ethanol extract of *S. macrophylla* seeds by employing a reverse-phase HPLC technique. Specificity, as defined by ICH, “the ability to assess the analyte for the presence of various components which may be expected to be present” (Guy 2014).

In this research, a closer examination of the chemical composition of *S. macrophylla* ethanolic extract at a concentration of 1 mg/mL was carried out. Swietenine, quantified at 27.539 μg/mg, and 3,6-O,O diacetyl swietenolide, quantified at 14.531 μg/mg, were identified using low-pressure gradient chromatography with reverse-phase chromatography. The identification of these two compounds in SMEE paves the way for discussing their potential roles in antidiabetic effects.

A different group of researchers successfully extracted swietenine from the chloroform fraction of *S. macrophylla* seeds, and this compound demonstrated remarkable (*p* < 0.01) activity comparable to human insulin (Maiti *et al.* 2009). Swietenine is recognised as a novel hypoglycaemic phytoconstituent (Kadota *et al.* 1990) that exhibits substantial peripheral glucose utilisation with an effect mimicking that of insulin (Maiti *et al.* 2009).

We have previously reported that the administration of 1,000 mg/kg b.w. of *S. macrophylla* petroleum ether extract (PE) effectively countered the increase in blood glucose levels after glucose ingestion and lowered blood glucose concentrations in normal rats, suggesting its potential to lower glucose levels. Additionally, it was observed that both in the presence and absence of insulin, isolated abdominal muscle showed significantly increased glucose uptake at PE concentrations of 1.0 mg/mL and 2.0 mg/mL (Hashim *et al.* 2013), indicating enhanced glucose utilisation by muscle tissue. Another research team, while investigating *S. macrophylla* seed methanol extract, reported a substantial reduction in fasting blood glucose levels in diabetic control rats compared to normal animals after a 12-day study (Maiti *et al.* 2009). In the search for the exact mechanism of action, it was revealed that the *S. macrophylla* seeds improved peripheral glucose utilisation (Maiti *et al.* 2009). Based on these findings, we explored the antidiabetic potential of *S. macrophylla* seed ethanol extract (SMEE) in GK Type 2 diabetic rats. These rats share similarities with the primary defects in beta cells and polygenic basis that are common in most Type 2 diabetes patients.

Our recent study has provided compelling evidence of the antihyperglycaemic effect of SMEE. Furthermore, we have demonstrated that SMEE exhibits oral glucose tolerance within a similar range as the positive control rats after 12 h of treatment administration. On day 15, although not statistically significant compared to the DC and GB groups, the blood glucose levels in the SMEE 500 group exhibited a decreasing trend and achieved considerably lower BGL within 60 to 120 min after the glucose challenge. Insulin levels showed no significant differences between the positive control and SMEE groups at all threetime points: 0 min, 30 min and 120 min. This indicates that SMEE exerts a similar impact on diabetic rats as the drug glibenclamide. Furthermore, on day 15, insulin levels in the SMEE group saw a slight decrease at 30 min post-glucose challenge, followed by an increase to the original level at 120 min post-glucose challenge. This could be attributed to the restorative process of pancreatic β-cells during the 14-day treatment with SMEE at 500 mg/kg. It is possible that the presence of metabolised glucose triggered β-cells to produce more insulin. The SMEE extract was found to improve the body weight of GK rats (*p* < 0.05), possibly due to enhanced glucose utilisation by the rats compared to the control groups DC and GB. This effect may be linked to the presence of 3,6-O,O diacetyl swietenolide and swietenine in SMEE, both known to promote GLUT4 translocation, facilitating increased glucose uptake in C2C12 cells (Lau *et al.* 2015).

In diabetic conditions, the surplus glucose in the bloodstream initiates a reaction with Hb, leading to the formation of HbA1c. The levels of HbA1c are directly correlated with blood glucose levels and the rate of glycation. Rats afflicted with diabetes, when treated with *S. macrophylla* ethanol extract, displayed a substantial reduction in HbA1c levels and a notable increase in Hb levels (Kalaivanan & Pugalendi 2011b; Rotruck *et al.* 1973). An imbalance in carbohydrate metabolism, often caused by a partial or complete lack of insulin, results in the diminished activity of several critical enzymes, including glucokinase, phosphofructokinase and pyruvate kinase. This imbalance hinders peripheral glucose uptake and promotes hepatic glucose synthesis. Among these enzymes, glucokinase plays a central role in the phosphorylation of glucose. It has been observed that the livers of Type 1 diabetic rats exhibit reduced glucokinase activity, likely linked to an insulin deficiency. Treatment with *S. macrophylla* and glibenclamide led to elevated insulin levels in STZ-induced Type 1 diabetic rats, improving glucokinase activity and thereby enhancing glucose utilisation. This cascade of events results in reduced blood sugar levels (Kalaivanan & Pugalendi 2011a). In our study, administering 500 mg/kg of *S. macrophylla* ethanolic extract significantly reduced fasting blood glucose levels in GK diabetic rats, indicating a similar mechanism of action involving the aforementioned enzymes. For future research endeavours, it is advisable to measure glucokinase levels in the livers of diabetic rats before and after treatment with *S. macrophylla*.

A study conducted by Dewanjee and their research team revealed that swietenine, administered at doses of 25 mg/kg and 50 mg/kg of b.w., led to a significant and dose-dependent reduction in fasting blood glucose levels in Type 2 diabetic rats (specifically, neonatal-streptozotocin diabetic Wistar albino rats) (Dewanjee *et al.* 2009). It is plausible that an increased dosage of SMEE, possibly around 1,000 mg/kg, would enhance insulin levels and, consequently, expedite the reduction of blood glucose levels in diabetic GK rats. Furthermore, conducting an OGTT test with a 1,000 mg/kg dose of SMEE following a glucose challenge would shed light on SMEE’s ability to regulate hyperglycemic conditions in the rats’ bloodstream. This additional research could provide valuable insights into the potential effectiveness of higher SMEE doses in managing blood glucose levels.

## CONCLUSION

At a dosage of 500 mg/kg b.w., SMEE exhibited notable antihyperglycaemic effects in Goto-Kakizaki Type 2 diabetic rats. These effects were characterised by a substantial reduction in fasting blood glucose levels that extended up to the 7th hour. Moreover, the results of the Oral Glucose Tolerance Test indicated that rats administered with SMEE displayed blood glucose and plasma insulin levels akin to those receiving the positive control, glibenclamide. Notably, rats in the SMEE group also exhibited lower initial fasting blood glucose levels on day-15 in comparison to day-1. The determination and measurement of swietenine and 3,6-O,O diacetyl swietenolide compounds in SMEE offered additional confirmation of the plant extract’s antihyperglycaemic properties, especially considering this was observed in a Type 2 diabetic animal model that closely simulates the effects seen in humans. In the future, the OGTT study could be designed to involve animals that are fed test extracts or compounds, followed by glucose administration to observe the plant extract’s/compound’s longer-term metabolic effects, beyond a 2-h period. Additionally, the impact of *S. macrophylla* extract (SMEE) and its active compounds, swietenine and 3,6-O,O-diacetyl swietenolide, on oxidative stress inhibition, glucose synthesis in the liver, liver function and liver toxicity can also be assessed.

## Figures and Tables

**Table 1:** Precision, intraday and interday analysis.

Analytes (Compound name)	Concentration (μg/mL)	Intraday precision			Interday precision		

		Mean	SD	%RSD	Mean	SD	%RSD
swietenine	1.56	1.53	0.01	0.62	1.37	0.31	22.84
	12.50	12.51	0.02	0.12	12.64	0.38	2.99
	50.00	50.80	0.19	0.38	54.04	5.37	9.94
	200.00	202.59	0.55	0.27	202.08	1.52	0.75

3,6-*O*,*O* diacetyl swietenolide	1.56	1.52	0.01	0.48	1.42	0.29	20.74
	12.50	12.49	0.03	0.23	12.62	0.36	2.88
	50.00	50.85	0.15	0.29	53.97	5.15	9.54
	200.00	202.37	0.45	0.22	202.06	1.65	0.82

**Table 2:** Mean percentage recoveries of the target analytes and the determined concentrations.

Analytes (Compounds name)	Conc.	Expected conc. (mg/mL) [C]	Spiked conc. (mg/mL) [D]	Recovery (%)

				[D/C] × 100
Swietenine	C1	29.10	26.06	89.56
	C2	40.04	37.86	94.55
	C3	77.54	78.63	101.41

3,6-O,O diacetyl swietenolide	C1	16.09	14.88	92.44
	C2	27.03	26.37	97.55
	C3	64.53	66.26	102.67

*Notes.* Conc. = concentration; Expected concentration (C) = unspiked concentration + calculated spike concentration.

**Table 3:** Body weight of rats before and after 14 days of treatment.

Groups	Description	Changes in body weights, BW (g)	

		Day 1	Day 15
NC	Negative control (4% Tween 80), normal	199.60 ± 13.33	211.90 ± 14.72
DC	Negative control (4% Tween 80), GK	204.00 ± 13.81	218.70 ± 11.10
GB	Positive control (Glibenclamide10mg/kg BW), GK	195.50 ± 14.49	208.20 ± 12.73
SMEE 500	Treated (SMEE 500mg/kg BW), GK	184.30 ± 12.00	201.70 ± 10.28*

*Notes.* Each value represents the mean ± s.e.m of six rats (n = 6).

* Significance (*p* < 0.05) compared to Day 1.

**Table 4:** Effects of oral glucose tolerance test (OGTT) on BGL on day 1, before the commencement of the respective treatments.

Treatment groups	0 min	30 min	60 min	90 min	120 min
NC, 4% Tween 80	5.18 ± 0.16^a^	9.07 ± 0.79^b^	7.62 ± 0.60^c^	5.75 ± 0.23^d^	5.53 ± 0.17^e^
DC, 4% Tween 80	9.88 ± 0.78	22.90 ± 1.64	19.95 ± 1.54	17.25 ± 1.21	15.82 ± 1.10
GB, 10 mg/kg	8.47 ± 0.13	21.97 ± 0.39	21.08 ± 0.74	18.57 ± 0.41	15.60 ± 0.77
SMEE 500, 500 mg/kg	9.88 ± 0.69	20.02 ± 0.74	16.92 ± 0.98	15.30 ± 1.64	12.48 ± 1.07

*Notes.* Each value represents the mean ± s.e.m of six rats (*n* = 6). NC: Normal rats + 4% Tween 80; DC: Type 2 diabetic GK rats + 4% Tween 80; GB: Type 2 diabetic GK rats + Glibenclamide 10 mg/kg (b.w.); SMEE 50: Type 2 diabetic GK rats + SMEE 500 mg/kg (b.w.). BGL significantly different (*p* < 0.05), compared to the DC untreated group of rats at (a) 0 min; (b) 30 min; (c) 60 min; (d) 90 min and (e) 120 min.

**Table 5:** Effects of oral glucose tolerance test (OGTT) on BGL on day 15, 14 days post-investigation.

Treatment groups	0 min	30 min	60 min	90 min	120 min
NC, 4% Tween 80	5.42 ± 0.26^a^	7.22 ± 0.29^b^	6.63 ± 0.39^c^	6.48 ± 0.51^d^	4.95 ± 0.57^e^
DC, 4% Tween 80	7.80 ± 0.33	21.27 ± 0.73	20.50 ± 0.54	18.58 ± 0.68	15.20 ± 0.51
GB, 10 mg/kg	7.65 ± 0.27	18.90 ± 1.79	19.72 ± 0.86	17.75 ± 0.67	14.15 ± 0.72
SMEE 500, 500 mg/kg	6.12 ± 0.30^a^	18.93 ± 1.45	19.60 ± 1.31	15.63 ± 1.17	13.45 ± 1.00

*Notes.* Each value represents the mean ± s.e.m of six rats (*n* = 6). NC: Normal rats + 4% Tween 80; DC: Type 2 diabetic GK rats + 4% Tween 80; GB: Type 2 diabetic GK rats + Glibenclamide 10 mg/kg (b.w.); SMEE 50: Type 2 diabetic GK rats + SMEE 500 mg/kg (b.w.). BGL significantly different (*p* < 0.05), compared to DC untreated group of rats at (a) 0 min; (b) 30 min; (c) 60 min; (d) 90 min and (e) 120 min.

**Table 6:** Effects of controls and SMEE on plasma insulin levels during OGTT in GK rats on days 1 and 15.

Groups	Description	Changes in insulin levels (μg/mL)					

		Day 1			Day 15		

		0 min	30 min	120 min	0 min	30 min	120 min
NC	Negative control (4% Tween 80), normal	0.38 ± 0.06	0.47 ± 0.05	0.46 ± 0.05	0.29 ± 0.03^a^	0.32 ± 0.04	0.28 ± 0.04^b^
DC	Negative control (4% Tween 80), GK	0.53 ± 0.08	0.48 ± 0.07	0.48 ± 0.06	0.49 ± 0.06	0.46 ± 0.05	0.48 ± 0.03
GB	Positive control (Glibenclamide 10 mg/kg b.w), GK	0.37 ± 0.07	0.36 ± 0.06	0.39 ± 0.07	0.55 ± 0.02	0.50 ± 0.03	0.47 ± 0.03
SMEE 500	Treated (SMEE 500 mg/kg b.w.), GK	0.51 ± 0.05	0.45 ± 0.05	0.48 ± 0.04	0.47 ± 0.05	0.45 ± 0.06	0.46 ± 0.07

*Notes:* Each value represents the mean ± s.e.m of six rats (*n* = 6). “a” represents BGL significantly different (*p* < 0.05), compared to 0 min of the DC untreated group on day 15. “b” represents BGL significantly different (*p* < 0.05), compared to 120 min of the DC untreated group on day 15.
